# Remote photonic detection of human senses using secondary speckle patterns

**DOI:** 10.1038/s41598-021-04558-0

**Published:** 2022-01-11

**Authors:** Zeev Kalyuzhner, Sergey Agdarov, Itai Orr, Yafim Beiderman, Aviya Bennett, Zeev Zalevsky

**Affiliations:** Faculty of Engineering and the Nano-Technology Center, Bar-Ilan University, 52900 Ramat-Gan, Israel

**Keywords:** Imaging, Sensors and probes, Software, Optical spectroscopy, Sensory processing, Lasers, LEDs and light sources, Optical techniques

## Abstract

Neural activity research has recently gained significant attention due to its association with sensory information and behavior control. However, the current methods of brain activity sensing require expensive equipment and physical contact with the tested subject. We propose a novel photonic-based method for remote detection of human senses. Physiological processes associated with hemodynamic activity due to activation of the cerebral cortex affected by different senses have been detected by remote monitoring of nano‐vibrations generated by the transient blood flow to the specific regions of the human brain. We have found that a combination of defocused, self‐interference random speckle patterns with a spatiotemporal analysis, using Deep Neural Network, allows associating between the activated sense and the seemingly random speckle patterns.

## Introduction

With its connection to sensory information and behavior control, neural activity research has recently received considerable attention. However, the current methods of brain activity sensing, involve expensive equipment and require physical proximity with the subject. Sensation is a physiological process involving sensory systems of a body responding to a stimuli and providing data for perception^1^. Human sensory systems are involved in daily activities, both consciously and unconsciously, and a study of the senses, especially their connection with brain activity, has been gaining in popularity in recent years. Brain activity analysis using electroencephalography (EEG) has received considerable attention^2–6^, particularly in relation to the five basic human senses: sight^2^, touch^7^, hearing^8,9^, smell^10–13^ and taste^14^. In addition to EEG, a number of optical techniques have also been employed for monitoring the human brain activity using image contrast analysis^15,16^ and cross-correlation based analysis of a laser speckle imaging^17^. While these methods mainly deal with a laser speckle image and its relation to temporal fluctuations, extracting semantic information from sensory activity is still lacking.

The temple area of the human head is located in front of the cerebral cortex and is not an optical quality surface. Therefore, when a rough surface is illuminated by a laser beam, the back scattered light forms secondary speckle patterns, which are possible to image clearly by a digital camera with defocused optics. Analysis of temporal changes in the spatial distribution of the speckle patterns can be related to nano-vibrations of the illuminated surface due to the hemodynamic process associated with the transient flow of the blood occurring during human brain activation^17^.

The speckle-based remote sensing has been used for the development of different biomedical applications, such as monitoring of the heart rate^18^, breathing^19^, blood pressure^20^, blood oximetry^21^, blood coagulation^22,23^, bone fractures^24^, melanoma^25^, and neural activity^17^. Prior methods for classifying speckle patterns used a single frame^26^ or full video frame-by-frame^21^ data obtained by averaging the model predictions on all frames of the video and providing a threshold for selecting the desired output. The prior classification methods used a Convolutional Neural Network (CNN) to encode data from a single frame. However, speckle pattern data recorded over successive periods could be characterized as time series data^27^. Due to this temporal dependency, we hypothesize that using a recurrent neural network architecture would provide improved results.

**Figure 1:**
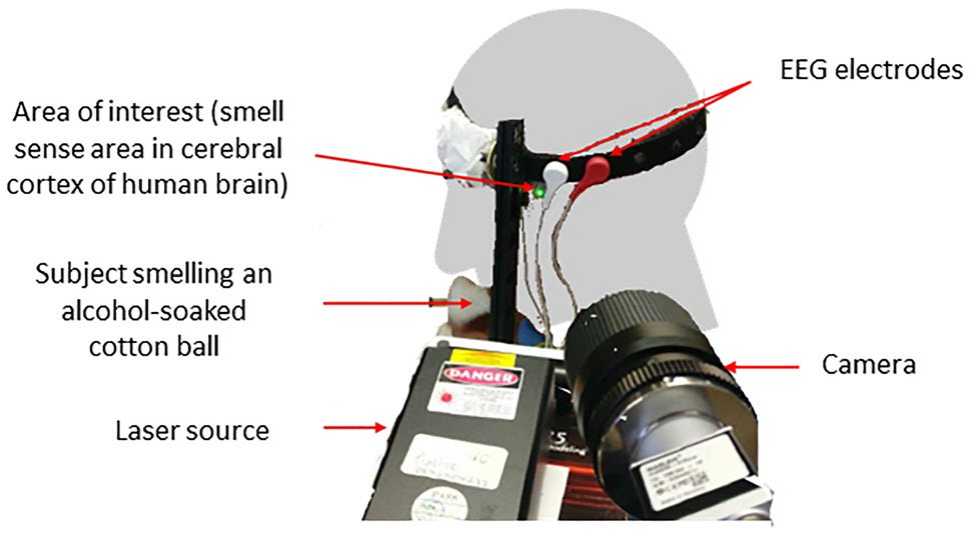
Experimental setup, including laboratory equipment and headset, for synchronized recording of speckle patterns and EEG signal obtained from the cerebral cortex of the human brain. While participating in the data acquisition process the subject smells an alcohol-soaked cotton ball, while the camera EEG are synchronized and are simultaneously recording the data.

We propose a new method for classification and detection of three basic senses: smell, taste, and hearing. Detection of senses is based on projecting a laser beam on the specific area of the human head being associated with the cerebral cortex activity, (see Fig. 1) and analyzing the recorded speckle patterns using DNN. To ascertain reliability of our approach, the results were compared with a synchronized and simultaneously recorded EEG, known to be an effective method for detecting brain activity related to human senses^2^. We trained an EEG-based DNN using the recorded EEG data and compared it to the results of the speckle-based DNN to find out conformity between the two approaches.

Our study could be of importance for patients suffering from stroke or cancer^28–31^ and experiencing irregularities in their basic senses, especially in taste and smell. The frequently occurring loss of taste and smell associated with COVID-19 is also noteworthy^32,33^. The olfactory neurons, which detect odors in the air and send signals to the brain, are one possible pathway for sensory loss^34^. Predicting sensory loss with relative simplicity and remotely can contribute to the discovery of COVID-19 virus carriers as well.

## Results

The experimental setup, shown in Fig. 1, comprises a laser, illuminating the temple area of a human head, and defocused high-speed camera, recording the reflected speckle patterns, an EEG device synchronized with the camera and a computer for the data processing. Eight healthy participants ranging in age from 29 to 74 have been tested in the two conditions: without and under stimulation of each sense.

**Figure 2:**
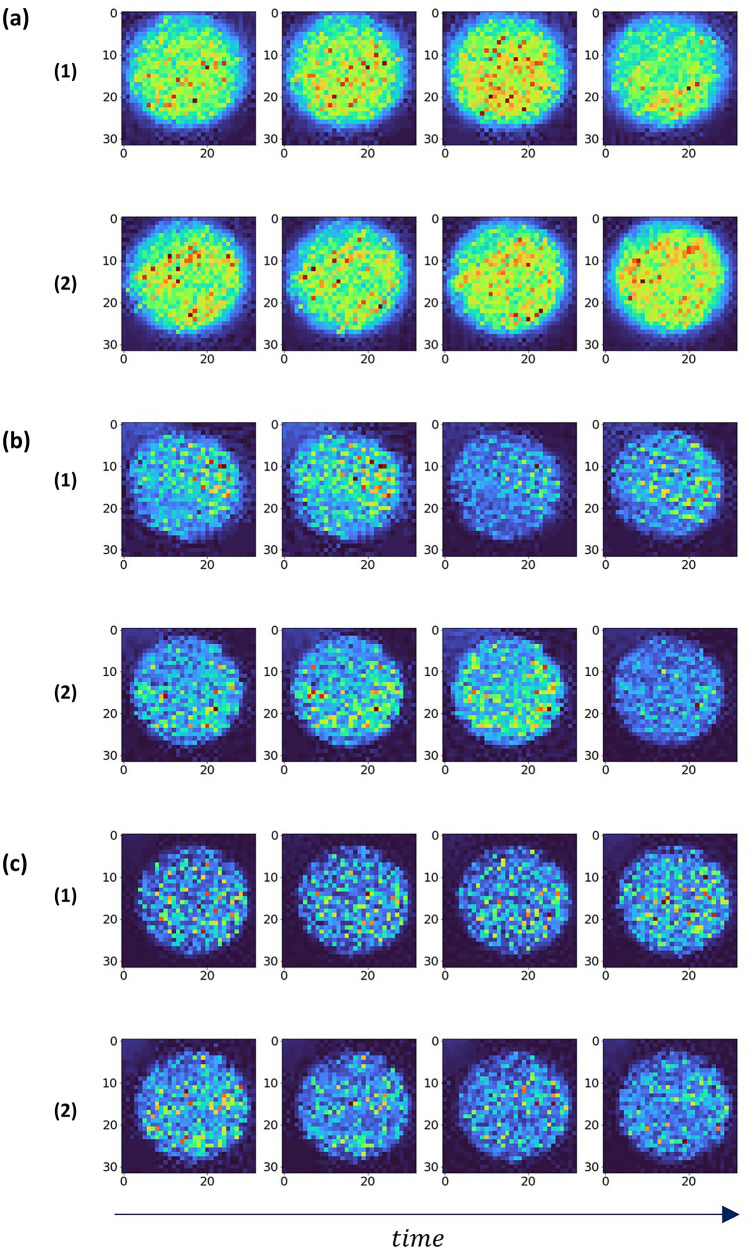
Sample frames from the dataset. (**a**) sample frames of smell sense. (**b**) sample frames of hearing sense. (**c**) sample frames of taste sense. For each sense (1) is representing frames of an active sense while (2) represents an inactive sense.

Figure 2 shows samples of the recorded speckle pattern for each sense in a consecutive timely related order from left to right. Figure 2a displays speckle patterns related to the sense of smell. Figure 2a1 represents the activated sense and Fig. 2a2 the inactive sense. Figure 2b displays the sense of taste: Fig. 2b1 represents the activated sense and Fig. 2b2 the inactive sense. Figure 2c displays the sense of hearing: Fig. 2c1 represents the activated sense and Fig. 2c2 the inactive sense.

Classification of the speckle patterns and its association to a specific brain activity was carried out using DNN. Results of our validation are given in Table 1, showing that our model achieved precision score of 92% and reached accuracy of 95%, while being faster and maintaining high recall of 98%. Table 1 also shows comparison of our model with the previous two methods for back scattered laser speckle patterns classification^21,26^ having accuracy of 82% and 89%.

**Table 1:** Speckle-based DNN comparison. Our method reaches an accuracy of 95% while maintaining a high recall of 98% in active-sense classification task. In an active-sense classification task, the full video approach achieved 89% accuracy and 93% precision. Our model takes 2 ms per batch to infer, while the single-frame method takes 4 ms, and the full-video method takes 830 ms.

Model	Classes	Accuracy (%)	Precision (%)	Recall (%)	F1 (%)	Inference time (ms)
Single speckle frame convnet	Sense	82	85	78	82	4
	No Sense		79	83		4
Full video speckle convnet	Sense	89	93	91	89	830
	No Sense		84	87		830
**Our model**	**Sense**	**95**	**98**	**92**	**95**	**2**
	**No Sense**		**92**	**98**		**2**

Significant values are in bold.

**Figure 3:**
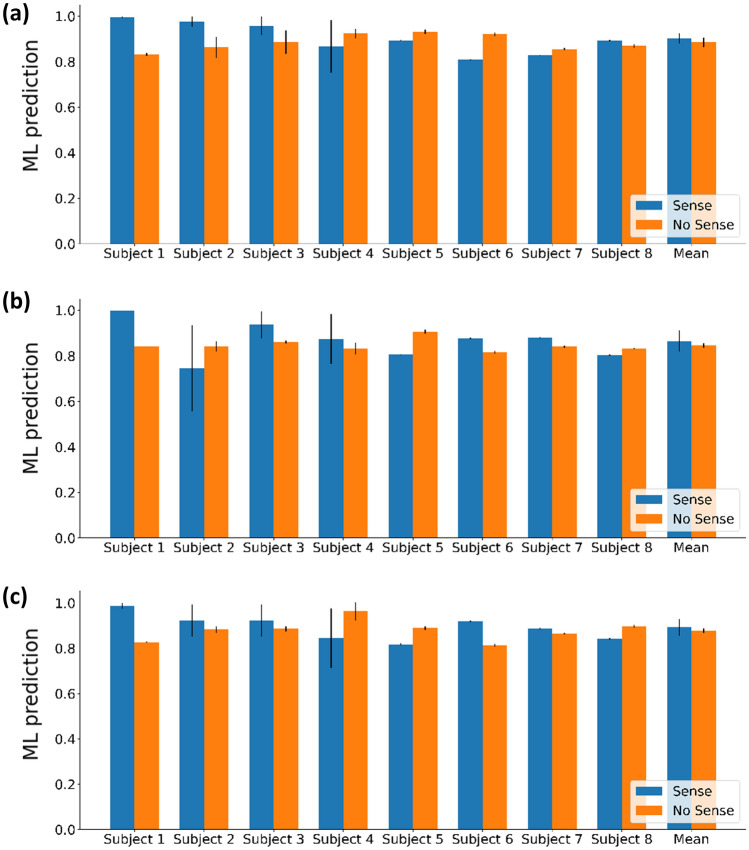
Speckle-based ML model sensory recognition for each subject and the average predictions for all participants for each of the three senses, including value margins for all tested subjects. Prediction details: First subject—male, age 29; Second subject—male, age 55; Third subject—male, age 74; Fourth subject—female, age 25; Fifth subject—male, age 49; Sixth subject—female, age 35; Seventh subject—male, age 50; Eight subject—male, age 55. The senses of smell (**a**), hearing (**b**), and taste (**c**) are all represented by Speckle-based DNN predictions. While the corresponding sense was active, the blue columns in each of the figures represents model predictions, while the orange columns represents model predictions when the sense was inactive.

Figure 3 presents the sensory recognition of our speckle based DNN for each subject and the average sensory recognition for each of the three senses, including value margins for all tested subjects. Figure 3a shows Speckle-based DNN predictions for the sense of smell, Fig. 3b—for the sense of hearing, and Fig. 3c—for the sense of taste. The blue columns in each figure represents the active sense predictions, while the orange columns—the inactive sense.

**Figure 4:**
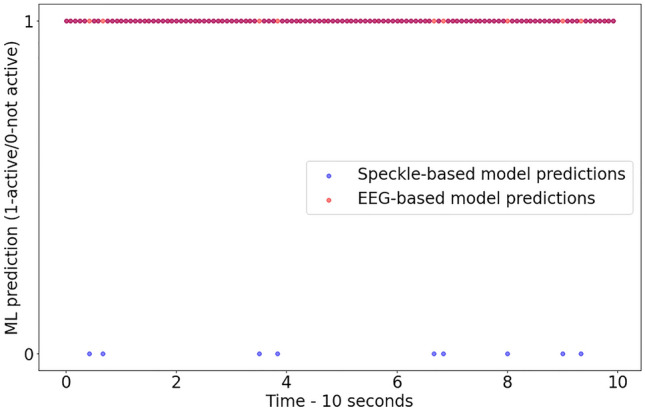
Comparison between the optical and EEG ML sense activity predictions. The predictions are based on simultaneous 10 s speckle and EEG inputs for one of the subjects while the sense of smell was active. The X-axis represents the time, and the binary EEG and speckle-based models’ predictions are shown on the Y-axis (0 when the sense is inactive and 1 when the sense is active).

The main benchmark according to which the results of our model are is verifiable is the sensing model based on the EEG input. Figure 4 shows our speckle-based model and the EEG model predictions for one tested subject. The speckle based and the EEG inputs were recorded simultaneously during 10 s period while the sense of smell was active. The speckle-based model predicted 120 input batches, each containing 64 frames recorded under 750 frames per second (FPS). In order to compare the EEG and the speckle-based model predictions, the percentage of matching values in the sample shown in Fig. 4was calculated. For the presented sample, the matching value is 92%, indicating that matching of EEG and our optical model are significant and high. For other tested participants the models matching value was in the range of 92–97%. Table 2 shows that the EEG-based DNN achieved an accuracy of 83% while maintaining a recall of 100% and precision of 75% in active-sense classification task.

**Table 2:** EEG-based DNN comparison. The EEG based model reaches accuracy of 83% while maintaining a recall of 100% and precision of 75% in active-sense classification task.

	Precision	Recall	F1-score	Accuracy
Sense	0.75	1.00	0.86	83
No-Sense	1.00	0.67	0.8	83

## Discussion

The current methods for sensing human brain activity, such as EEG and MRI, require significant resources , expensive equipment and close proximity or physical contact with a patient. We propose a new photonic-based remote monitoring method for detection of human senses by combining a deep learning approach with spatiotemporal analysis of defocused self-interference random speckle patterns reflected from the specific temple area of the human head. This work provides further evidence for the hypothesis that physiological processes associated with the hemodynamic activity of the brain due to stimulation of the cerebral cortex by different senses, could be identified by remote monitoring of nano-vibrations produced by the transient blood flow to the specific regions of the head.

The precise distribution of blood flow, as well as the resultant speckle patterns, are both subject-dependent. However, we do not measure or analyze the speckle pattern itself, but rather the temporal changes it exhibits. The differences, or changes, are entirely the result of blood activity that began in the particular cortex when the particular sense is activated. We hypothesize that this assumption will hold true for any and all participants since human structural component is essentially comparable in terms of the relevant cortexes located in similar areas of the brain.

Temporal changes in the spatial distribution of the random speckle patterns can affect precision of the single frame method : the single frame might not represent whole recording session^26^.

One major limitation of the secondary speckle patterns classification using full video frame-by-frame method is the noise added due to the multiplicity of frames required to obtain prediction^21^. Namely, the first and the last recorded frames could contain irrelevant information due to subject’s behavior and unintentional head movements.

The two prior methods are based on a convnet model, which does not consider temporal dependency related to speckle pattern signal.

Our model, compared to the previously applied methods, includes a ConvLSTM layer that considers temporal dependency being found in our data in addition to the image processing capabilities of the convolution layers.

The underlying physiological processes of the human brain activity are time-dependent, hence, the ConvLSTM based model allows to learn important features related to the hemodynamic activity due to activation of the cerebral cortex of the human brain.

Figure 3 shows that sensory activity detection can be classified using learning-based methods. No significant difference in the model identification was found between the different types of senses or subjects, since in all cases the model input is expressed through the nano‐vibrations associated with neural activity due to activation of the cerebral cortex of the subject's brain.

The comparison between the photonic and EEG-methods for human senses detection and classification shows high conformity between EEG and our speckle-based model.

## Methods

### Experimental setup

The experimental setup comprises a green laser (770 µW, 532 nm), Basler saA1300-200um area scan camera with defocused optics to generate and capture the speckle patterns, EEG electrodes, OpenBCI EEG headband with Ganglion bio-sensing 4-channels boar and a computer. The video and EEG were synchronized to record brain signals simultaneously.

Data was collected from Eight healthy participants, ages 29–74, in a shuttered and controlled laboratory environment to prevent background noises. Each subject was seated on a distance of 50 cm from the camera, as shown in Fig. 1. The subject’s head was restrained in a headset equipped with a protective gear for the purpose of directing the left side of the head to the sensor and mitigating involuntary head movements. Each subject’s smell, taste and hearing sense-related brain activity was recorded in two conditions. First, in the state of rest without sensory stimulation. Second, under stimulation of each sense by performing a relevant action. In order to stimulate the sense of smell the subject smelled an alcohol-soaked cotton ball; the sense of taste was stimulated with sweet chocolate and the sense of hearing with a continuous constant-frequency noise.

A high-speed digital camera with defocused optics recorded the temporal changes of the speckle images during 10 s sampling for each test. The frame rate was set to 750 FPS and spatial resolution of 32 × 32 pixels. Data collection was performed on separate dates, with each subject recorded five times in one continuous session. The dataset contained roughly 240,000 frames where each subject's video contained a unique identification, including the subject's ID, the duration of the measurement, the sense type, and a binary sign symbolizing activity or inactivity of the sense.

The videos from different recording days were sub-divided into training and test datasets prior to subdividing them into specific frames. Data for all tested subjects was included in the training and test sets, preventing any mixing between the training and test datasets, which could have occurred with a simple random split.

Quantitative assessment and comparison of our proposed method used the metrics provided in Eqs. (2–5) with TP: True Positive, TN: True Negative, FP: False Positive and FN: False Negative, calculated pixelwise by the logical operators given in Eq. (1).

1 $$\begin{array}{*{20}c} {TP_{i} = \left( {x_{i} = = 1} \right)\;\& \;\left( {y_{i} = = 1} \right)} & {TN_{i} = \left( {x_{i} = = 0} \right)\;\& \;\left( {y_{i} = = 0} \right)} \\ {FP_{i} = \left( {x_{i} = = 1} \right)\;\& \;\left( {y_{i} = = 0} \right)} & {FN_{i} = \left( {x_{i} = = 0} \right)\;\& \;\left( {y_{i} = = 1} \right)} \\ \end{array}$$

2 $$accuracy = \frac{1}{n}\mathop \sum \limits_{i = 1}^{n} \frac{{TP_{i} + TN_{i} }}{{TP_{i} + TN_{i} + FP_{i} + FN_{i} }}$$

3 $$precision = \frac{1}{n}\mathop \sum \limits_{i = 1}^{n} \frac{{TP_{i} }}{{TP_{i} + FP_{i} }}$$

4 $$recall = \frac{1}{n}\mathop \sum \limits_{i = 1}^{n} \frac{{TP_{i} }}{{TP_{i} + FN_{i} }}$$

5 $$F_{1} = \frac{1}{n}\mathop \sum \limits_{i = 1}^{n} \frac{{2TP_{i} }}{{2TP_{i} + FN_{i} + FP_{i} }}$$

where the tuple ${(x}_{i},{y}_{i})$ is the model prediction and label for sample i.

The Institutional review board of Bar-Ilan University provided the ethics approval for the study. All participants provided informed consent for participation in the study. The experiments were carried out in accordance with relevant guidelines and regulations. Although deconstructed for lab optimization purposes, the device is entirely laser safe, tissue safe, etc., as previously obtained from international regulators.

### Model

**Figure 5:**
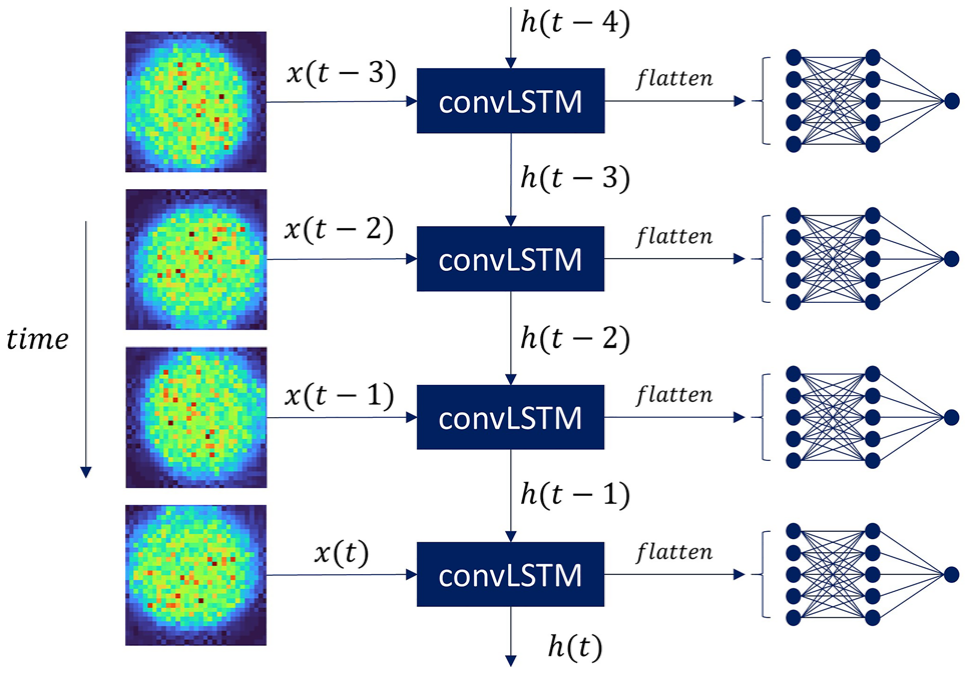
DNN classifier architecture. A visualization of the model in operation on a speckle input signal is shown. With state-to-state kernels of size 3 × 3, the ConvLSTM single layer network contains 64 hidden states and input-to-state for 64 input frames. Since the required model's prediction is a binary classification task, we concatenated all forecasting network's states and fed them into a 256-unit dense with ReLU activation, which speeds up our model's training phase by reducing the gradient of the computational process. To produce the final prediction, we added a dense layer with two units on top of the model.

We propose to process the sequential speckle images by using a 2D ConvLSTM layer^35^ consisting of the LSTM layer with internal matrix multiplications along with 2D convolution operations. In our configuration, the speckle based data passes through the ConvLSTM cells and retains the original input dimensions instead of being projected onto a 1D feature vector, as seen in Fig. 5.

The main equations of the ConvLSTM layer^35^ are given below (Eq. 6):

6 $$\begin{aligned} i_{t} & = \sigma \left( {W_{xi} *X_{t} + W_{hi} *H_{t - 1} + W_{ci} \circ C_{t - 1} + b_{i} } \right) \\ f_{t} & = \sigma \left( {W_{xf} *X_{t} + W_{hf} *H_{t - 1} + W_{cf} \circ C_{t - 1} + b_{f} } \right) \\ o_{t} & = \sigma \left( {W_{xo} *X_{t} + W_{ho} *H_{t - 1} + W_{co} \circ C_{t} + b_{o} } \right) \\ C_{t} & = f_{t} \circ C_{t - 1} + i_{t} \circ \tanh \left( {W_{xc} *X_{t} + W_{hc} *H_{t - 1} + b_{c} } \right) \\ H_{t} & = o_{t} \circ \tanh \left( {c_{t} } \right) \\ \end{aligned}$$

where ∗ denotes the convolution operator; ◦ is the Hadamard product $; {{\mathrm{X}}_{1}\dots \mathrm{X}}_{\mathrm{t}}$ represent the model input$; {\mathrm{C}}_{1}\dots {\mathrm{C}}_{\mathrm{t}}$ are the ConvLSTM cell outputs $; {\mathrm{H}}_{1}\dots {\mathrm{H}}_{\mathrm{t}}$ are the hidden states$; {\mathrm{o}}_{\mathrm{t}},{\mathrm{f}}_{\mathrm{t}},{\mathrm{i}}_{\mathrm{t}}$ are the ConvLSTM layer's 3D tensors, with the last two dimensions being spatial dimensions.

The ConvLSTM is defined by the inputs, past states of its local neighbors and the potential state of a certain cell in the grid. Before implementing the convolution operation, padding is required to ensure that the states and the inputs have the same number of rows and columns.

For the ConvLSTM model, the patch size to 1 × 1 was set to represent each 32 × 32 frame by a 32 × 32 × 3 tensor. The ConvLSTM single layer network contained 64 hidden states and input-to-state for 64 input frames, with state-to-state kernels of size 3 × 3. The output from the ConvLSTM encoder was directed into a fully connected, binary classification head, which contained 256-unit and ReLU activations.

Training was implemented in TensorFlow. Additional implementation details include binary cross-entropy as a loss function and dropout to reduce overfitting. We used the Adam optimizer with beta_1 = 0.9, beta_2 = 0.999. The batch size was 64 and learning rate—− 0.001. Training was performed on a single 1080Ti GPU and took roughly 20 epochs to converge.

### EEG data classification

In order to verify the validity of the proposed method, the EEG and the speckle pattern recordings were synchronized. To perform classification of the EEG signal, we used a CNN with three 1D-Conv layers with a ReLU activation function and a 1D-MaxPooling operation followed by two fully connected layers^36^. No further preprocessing was used prior to the EEG-based model.

Additional implementation details include binary cross-entropy used as a loss function and the Adam optimizer. Training was performed on a single 1080Ti GPU and took roughly 10 epochs to converge.

### Comparison of the validation methods

Prior methods for analyzing speckle patterns required a single frame^26^ or the entire video frame-by-frame^21^, achieved by averaging the model predictions across all frames of the video and giving a threshold for attempting to pick the desired outcome. Prior classification techniques utilized a Convolutional Neural Network (CNN) to encode data from a single frame.

TensorFlow implemented those two approaches for training. Additional implementation details include binary cross-entropy as a loss function and dropout to reduce overfitting. We used the Adam optimizer with beta_1 = 0.9, beta_2 = 0.999. The batch size was 32 and learning rate—0.001. Training was performed on a single 1080Ti GPU and took roughly 40 epochs to converge. Table 1 shows the validation results, which demonstrate that our method achieved a precision score of 92%, an accuracy score of 95%, while being faster and maintaining a high recall of 98%. Table 1 also compares our model to the previous two methods for back scattered laser speckle pattern classification, which had accuracy of 82 and 89%, respectively.

## Conclusions

This paper presents a new speckle based photonic method for remote monitoring and detection of the three basic human senses: smell, taste, and hearing.

Base of the method is a combination of spatiotemporal analysis of defocused self-interference random speckle patterns reflected from the specific temple area of the head with a deep learning approach.

The study provides further evidence for the hypothesis that physiological processes associated with hemodynamic brain activity due to stimulation of the cerebral cortex by different senses could be identified by remote monitoring of nano-vibrations produced by transient blood flow to the specific regions of the head. The developed DNN showed high accuracy in classifying active and inactive senses.

Our method offers an alternative and much simpler solution for detecting specific brain activity which otherwise require significant resources (for example EEG or MRI devices). Furthermore, future development of our method could allow remote monitoring and evaluation of human brain activity on a large scale due to the low cost and flexibility of the system.

## Data Availability

The data generated to support the findings of this study are available from the corresponding author upon reasonable request.

## Code availability

The code is available at https://github.com/zeevikal/senses-speckle.

## References

[1] Proctor, R. W. & Proctor, J. D. Sensation and perception. Handbook of human factors and ergonomics, 55—90 (2006).

[2] Alarcão SM, Fonseca MJ (2019). Emotions recognition using EEG signals: A survey. IEEE Trans. Affect. Comput..

[3] Zhuang, X., Sekiyama, K. & Fukuda, T. Evaluation of human sense by biological information analysis. In *20th Anniversary MHS 2009 and Micro-Nano Global COE - 2009 International Symposium on Micro-NanoMechatronics and Human Science* 74–79. 10.1109/MHS.2009.5352071 (2009).

[4] Lee M, Cho G (2009). Measurement of human sensation for developing sensible textiles. Hum. Factors Ergon. Manuf..

[5] Park KH (2019). Evaluation of human electroencephalogram change for sensory effects of fragrance. Ski. Res. Technol..

[6] Fukai H, Tomita Y, Mitsukura Y (2013). A design of the preference acquisition detection system using the EGG. Int. J. Intell. Inf. Syst..

[7] Nakamura T, Tomita Y, Mitsukura Y (2011). A method of obtaining sense of touch by using EEG. Inf..

[8] Zoefel B, VanRullen R (2016). EEG oscillations entrain their phase to high-level features of speech sound. Neuroimage.

[9] Christensen CB, Harte JM, Lunner T, Kidmose P (2018). Ear-EEG-based objective hearing threshold estimation evaluated on normal hearing subjects. IEEE Trans. Biomed. Eng..

[10] Lorig TS (2000). The application of electroencephalographic techniques to the study of human olfaction: A review and tutorial. Int. J. Psychophysiol..

[11] Martin GN (1998). Human electroencephalographic (EEG) response to olfactory stimulation: Two experiments using the aroma of food. Int. J. Psychophysiol..

[12] Saha A, Konar A, Chatterjee A, Ralescu A, Nagar AK (2014). EEG analysis for olfactory perceptual-ability measurement using a recurrent neural classifier. IEEE Trans. Hum. Mach. Syst..

[13] Saha A, Konar A, Rakshit P, Ralescu AL, Nagar AK (2013). Olfaction recognition by EEG analysis using differential evolution induced Hopfield neural net. Proc. Int. Jt. Conf. Neural Netw..

[14] Park C, Looney D, Mandic DP (2011). Estimating human response to taste using EEG. Proc. Annu. Int. Conf IEEE Eng. Med. Biol. Soc. EMBS.

[15] Boas DA, Dunn AK (2010). Laser speckle contrast imaging in biomedical optics. J. Biomed. Opt..

[16] Jiang M (2012). Dynamic imaging of cerebral blood flow using laser speckle during epileptic events. Biomed. Opt. Biomed..

[17] Ozana N (2020). Remote photonic sensing of cerebral hemodynamic changes via temporal spatial analysis of acoustic vibrations. J. Biophoton..

[18] Zalevsky Z (2009). Simultaneous remote extraction of multiple speech sources and heart beats from secondary speckles pattern. Opt. Express.

[19] Lengenfelder B (2019). Remote photoacoustic sensing using speckle-analysis. Sci. Rep..

[20] Golberg M, Ruiz-Rivas J, Polani S, Beiderman Y, Zalevsky Z (2018). Large-scale clinical validation of noncontact and continuous extraction of blood pressure via multipoint defocused photonic imaging. Appl. Opt..

[21] Kalyuzhner Z, Agdarov S, Bennett A, Beiderman Y, Zalevsky Z (2021). Remote photonic sensing of blood oxygen saturation via tracking of anomalies in micro-saccades patterns. Opt. Express.

[22] Ozana N (2015). Demonstration of a remote optical measurement configuration that correlates with breathing, heart rate, pulse pressure, blood coagulation, and blood oxygenation. Proc. IEEE.

[23] Beiderman Y (2010). Remote estimation of blood pulse pressure via temporal tracking of reflected secondary speckles pattern. J. Biomed. Opt..

[24] Bishitz Y (2015). Noncontact optical sensor for bone fracture diagnostics. Biomed. Opt. Express.

[25] Ozana N (2016). Remote optical configuration of pigmented lesion detection and diagnosis of bone fractures. Photonic Ther. Diagn. XII.

[26] Kalyzhner Z, Levitas O, Kalichman F, Jacobson R, Zalevsky Z (2019). Photonic human identification based on deep learning of back scattered laser speckle patterns. Opt. Express.

[27] Connor JT, Martin RD, Atlas LE (1994). Recurrent neural networks and robust time series prediction. IEEE Trans. Neural Netw..

[28] Banerjee, T. K., Roy, M. K. & Bhoi, K. K. Is stroke increasing in India—preventive measures that need to be implemented. *J. Indian Med. Assoc.* **103** 162, 164, 166 passim–162, 164, 166 passim (2005). 16173293

[29] Green TL, McGregor LD, King KM (2008). Smell and taste dysfunction following minor stroke: a case report. Can. J. Neurosci. Nurs..

[30] Penry WHTRJP, Kiffin J (1983). Complex partial seizures clinical characteristics and differential diagnosis. Handb. Park. Dis. Fifth Ed..

[31] Nguyen MQ, Ryba NJP (2012). A smell that causes seizure. PLoS ONE.

[32] Tong JY, Wong A, Zhu D, Fastenberg JH, Tham T (2020). The prevalence of olfactory and gustatory dysfunction in COVID-19 patients: A systematic review and meta-analysis. Otolaryngol. Head Neck Surg. (United States).

[33] Walker A, Pottinger G, Scott A, Hopkins C (2020). Anosmia and loss of smell in the era of covid-19. BMJ.

[34] Cooper KW (2020). COVID-19 and the chemical senses: Supporting players take center stage. Neuron.

[35] Shi, X. *et al.* Convolutional LSTM network: A machine learning approach for precipitation nowcasting. *Adv. Neural Inf. Process. Syst.* 802–810 https://arxiv.org/abs/1506.04214 (2015).

[36] Craik A, He Y, Contreras-Vidal JL (2019). Deep learning for electroencephalogram (EEG) classification tasks: A review. J. Neural Eng..

